# Variation in self-compatibility among genotypes and across ontogeny in a self-fertilizing vertebrate, *Kryptolebias marmoratus*

**DOI:** 10.1098/rspb.2025.0919

**Published:** 2025-08-13

**Authors:** Jennifer D. Gresham, Anna Clark, Chloe M. T. Keck, Alexis E. Longmire, Abye E. Nelson, Haylee Quertermous, Ashley B. White, Ryan Earley

**Affiliations:** ^1^ Biology Department, Pennsylvania State University, Dunmore, PA 18512, USA; ^2^ Department of Biological Sciences, The University of Alabama System, Tuscaloosa, AL 35487, USA; ^3^ Department of Biological Sciences, University of South Carolina, Columbia, SC 29208, USA; ^4^ University of North Carolina, Institute of Marine Sciences, Morehead City, NC 28557, USA; ^5^ Department of Anatomy, University of Otago, Dunedin, Otago 9016, New Zealand; ^6^ The University of Alabama at Birmingham, Birmingham, AL 35294, USA

**Keywords:** self-compatibility, mixed mating, age, life history, *Kryptolebias marmoratus*, genotypic variation

## Abstract

Mixed-mating strategies can maximize the benefits and limit the costs of both self-fertilization and outcrossing. In addition to ecological conditions and population dynamics, the economics of mixed mating are determined by individual self-compatibility, i.e. the proportion of self-fertilization events that result in viable offspring. In gynodioecious (hermaphrodites and females) and androdioecious (hermaphrodites and males) species, self-compatibility of hermaphrodites dictates the reproductive potential of the other sex and can exert strong selection on maintenance of the non-hermaphroditic sex. Mangrove rivulus fish populations are androdioecious, and males result from hermaphrodites changing sex. Hermaphrodites overwhelmingly reproduce through internal self-fertilization, but occasionally oviposit unfertilized eggs, which males can fertilize externally. We tested the hypotheses that self-compatibility and fecundity would vary with age and as a function of genotypic variation in propensities for sex change. We reveal that fecundity and self-compatibility vary within individuals across ontogeny and among genotypes with different propensities to change sex. Hermaphrodites from genotypes that frequently change sex were significantly less fecund and self-compatible than hermaphrodites from genotypes that rarely change sex. These differences in self-compatibility and fecundity have the potential to drive mating strategy evolution in mangrove rivulus, specifically the fitness of males and associated spatiotemporal variation in sex ratios within and among populations.

## 1. Introduction

As a reproductive strategy, self-fertilization has many benefits. It overcomes the ‘cost of males’ accrued by dioecious, sexually reproducing organisms, wherein only half the population can produce offspring compared with the entire population in asexual or self-fertilizing species [1,2]. Self-fertilization also assures reproduction when pollinators and/or mates are scarce [3–6], eliminates physical damage associated with mating and/or sexually transmitted parasites or pathogens [7,8], may help in purging deleterious mutations (reviewed in [9]) and maintains well-adapted genotypes in stable environments [10–12]. However, selection for selfing is often counteracted by selection for outcrossing. Benefits of outcrossing include combatting inbreeding depression by preventing accumulation of deleterious alleles in single genotypes and maintaining heterozygotes that may be more fit than homozygous individuals [13,14]. Ultimately, outcrossing represents a bet-hedging strategy because it increases the variability of offspring fitness against a changing abiotic environment or against coevolving parasites, predators and competitors [13,15–18].

Many plant and invertebrate species have evolved a mixed-mating strategy of selfing and outcrossing [19–23]. Mixed mating allows individuals to reap the rewards of both strategies as biotic and abiotic factors change the fitness landscape across space and time. Because the fitness landscape is variable, self-compatibility (the proportion of self-fertilization events resulting in viable offspring) also is expected to vary both within and among populations owing to changes in the magnitude and/or type of selection for and against selfing/outcrossing [24]. Plant populations that are often mate- and/or pollinator-limited at the range edge or in ephemeral habitats have increased self-compatibility compared with other populations, such as for the small angiosperm *Leavenworthia alabamica* [25]. Populations found near the centre of the range and in more permanent habitats are much more likely to be self-incompatible [26]. When among-individual variation in self-compatibility is heritable, it provides the genetic and phenotypic foundation upon which selection can drive evolutionary change in mating strategies. There are multiple examples of heritable variation in the self-compatibility of plants, including creeping bellflower, *Campanula rapunculoide* [26], and Carolina horsenettle, *Solanum carolinense* [27]. Genetic variation in self-compatibility, along with environmental variability, can drive among-individual variation in mating strategy because, together, they alter the economics of selfing and outcrossing. Such variation then serves as fodder for future selection to direct changes in the magnitude and direction of mating strategy evolution.

Genetic and environmental variability can also produce variation in self-compatibility across ontogeny, for example as a flexible response to environmental conditions, where individuals become more self-compatible with increasing age. Ontogenetic shifts in self-compatibility could result from selection favouring a ‘waiting’ strategy for outcrossing opportunities when individuals are young, presumably to derive benefits from variable and potentially fitter progeny but also to assure some reproduction even if mates and/or pollinators are limited (e.g. plants: *C. rapunculoide* [26] and *S, carolinense* [27]; cestodes*: Schistocephalus solidus* [28]; and snails: *Physa acuta* [29]).

In androdioecious species, populations consist of selfing hermaphrodites and males, and variation in self-compatibility should also result in variation in the proportion of males within a population. Populations consisting of individuals with lower self-compatibility might be expected to maintain a higher proportion of males, as the ‘cost of males’ is decreased by more outcrossing opportunities. Hypotheses concerning the evolution of self-compatibility and mixed mating have been examined in many plant and invertebrate systems [25,30,31], but not in a vertebrate species. Here, we present evidence for individual variation in self-compatibility in one of only two vertebrates known to utilize a mixed-mating strategy, the mangrove rivulus fish (*Kryptolebias marmoratus* (this study), *K. hermaphroditus*; see also the ‘central clade’ [32–34]).

**Figure 1.**
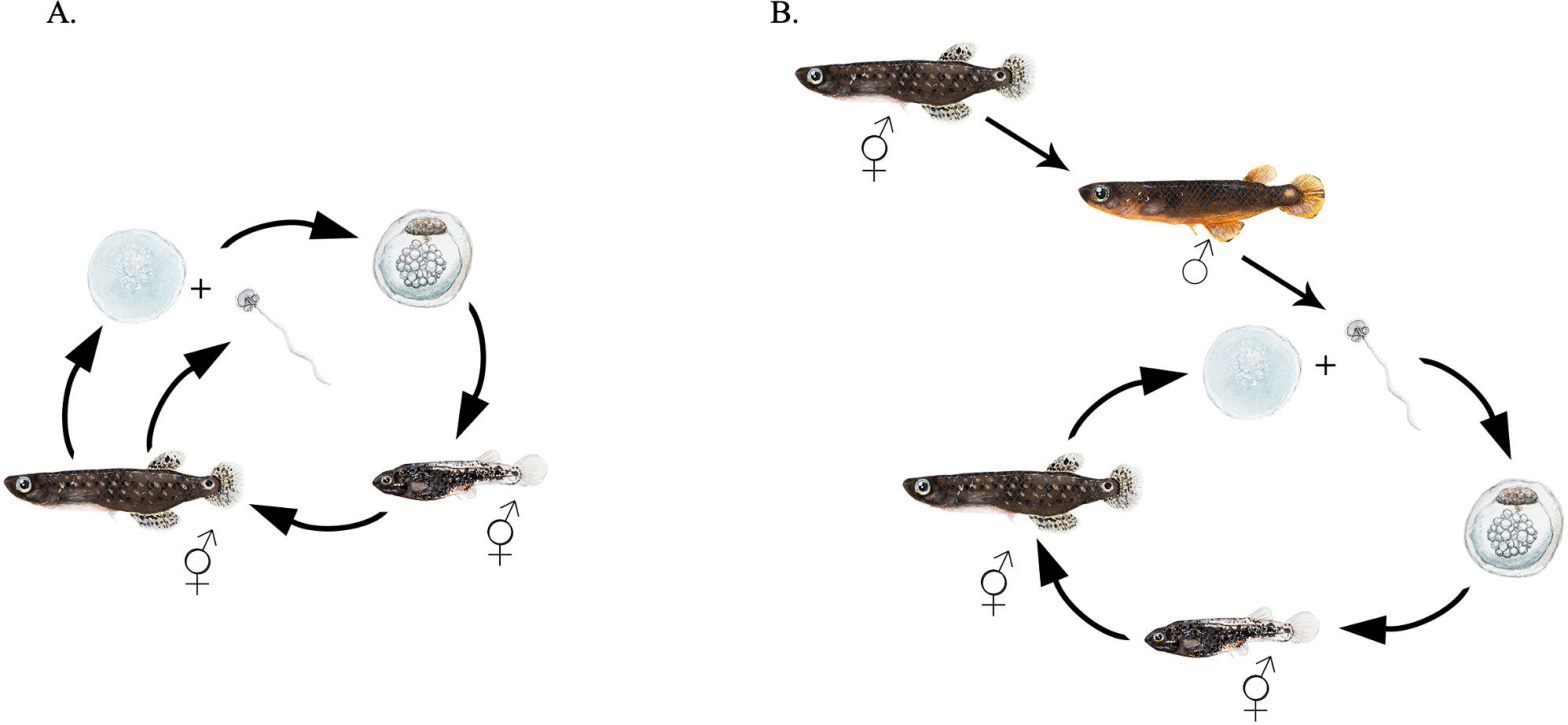
*Kryptolebias marmoratus* (‘rivulus’) life cycles. (A) Life cycle of individuals that remain self-fertilizing hermaphrodites, showing egg and sperm. (B) Life cycle of individuals that change sex to male after sexual maturity and become obligate outcrossers (egg from hermaphrodite and sperm from male). Note that some individuals self-fertilize as hermaphrodites before sex change. Details of morphological changes (colour and ocellus) with sex change found in [39]. The figure is hand-illustrated (watercolour and pencil) by Brooke M. Fitzwater. Egg illustrations were inspired by Mourabit *et al.* [40], the sperm illustration by Kweon *et al.* [41] and the hatchling illustration by photographs from Johanna Nelson.

Mangrove rivulus (hereafter ‘rivulus’) is a small euryhaline killifish that inhabits high-elevation (i.e. relatively far from the coast) mangrove forests of Florida, the Bahamas and Central America. Populations consist primarily of self-fertilizing hermaphrodites with varying proportions of males (no females) [35,36]. Rates of outcrossing and selfing also vary among populations and correlate with the proportion of males in the population; outcrossing rates increase as the proportion of males increases [37,38]. Since self-fertilization happens internally, males can only achieve reproductive success if/when hermaphrodites lay unfertilized eggs (figure 1). There is no evidence that hermaphrodites outcross with each other [42].

Rivulus males result from sex change, during which ovarian tissue is resorbed and testicular tissue proliferates, and after which selfing hermaphrodites become obligate male outcrossers (figure 1) [43]. In laboratory conditions, sex change can occur when fish are housed in isolation or socially. We do not have any evidence that population size or population sex ratio affects which and how many fish change sex to male in laboratory conditions. Sex change also takes place at variable times after sexual maturity [43] but is affected by water temperature [44–46] and artificial short-day seasons [43]. Importantly, there is genetic variation in the propensity to change sex; some genotypes change sex to male consistently in controlled laboratory conditions, while individuals of other genotypes rarely, if ever, change sex [45,47].

Hermaphrodites fertilize most of their own eggs (>94% [43,48]), giving males very limited opportunities for reproductive success because they must also find viable eggs to fertilize. While individuals that change sex to male are significantly more likely to survive environmental challenges than those that remain hermaphrodite, they are also far less likely to lay any eggs before sex change occurs [47]. This significant decrease in reproductive assurance begs the question of how sex-changing alleles are successfully passed to the next generation.

Broadly, we are interested in understanding the mechanisms that lead to variation in sex ratio and outcrossing rates among rivulus populations. More specifically, we aim to determine how sex-change alleles are maintained in some populations but seem to be absent (or at least not expressed) in others. Our overall hypothesis was that there would be variation in individual self-compatibility, which we define as the proportion of self-fertilized eggs that yield viable offspring. Individual self-compatibility, along with environmental/social context, might determine the *per capita* cost of changing sex. For instance, a highly self-compatible individual (that fertilizes most of its eggs and yields a high proportion of viable offspring) would pay a greater reproductive cost by changing sex than a less self-compatible individual. However, the cost of sex change may be significantly reduced if a sex-changed male finds itself in a population of hermaphrodites with low self-compatibility that lay more unfertilized eggs.

To understand the individual costs of sex change, how individual self-compatibility variation affects fitness within populations and how sex-change alleles might persist in populations, we need to document variation in self-compatibility in a controlled experiment. Therefore, we explored how self-compatibility varies with genotypic variation in the propensity for sex change (PFSC) and with age. As fecundity is also a trait that greatly determines individual fitness, we also documented relationships between fecundity and both PFSC and age.

We hypothesized that the proportion of fertilized eggs (a measure of self-compatibility) would vary with age and/or the genotype’s PFSC. First, we predicted that self-compatibility would increase with age. Cole & Noakes [49] reported histological evidence that ovarian tissue matures before spermatogenic tissue. We thus predicted that there would be a period immediately after sexual maturity when oviposition of unfertilized eggs would be more likely. Second, we predicted that fecundity and/or self-compatibility would be greater in high PFSC genotypes for two non-mutually exclusive reasons. First, while we do not have estimates of male fitness in rivulus populations, it is likely to be very low after sex change (>94% of eggs are self-fertilized [43]). Thus, high fecundity prior to sex change might compensate for any lost reproductive success experienced as a male, potentially resulting in the correlated evolution of sexual plasticity and reproductive investment. Second, while most rivulus populations harbour significant genetic variation (despite selfing [50]), multiple representatives of each genotype coexist in space and time [51]. Thus, the fitness of each genotype is best thought of as an average across all individuals sharing that genotype. Some individuals will remain hermaphrodites, and others might change sex to male; across 359 genetically distinct lineages, PFSC ranges from 0 to 87.5% of the individuals within a genotype [47]. High fecundity and self-compatibility of individuals that remain hermaphrodites might therefore compensate for any decrement in fitness associated with other genotype representatives that change sex. In this case, high and low PFSC genotypes might have equal lifetime fitness, which ultimately maintains variation in sex change-related alleles in the population.

## 2. Methods

### (a) Study design and procedures

We measured fecundity and self-compatibility in 213 individual fish, descended from wild-caught individuals, representing 100 genetically distinct genotypes (determined via microsatellite genotyping using 32 loci [52,53], with most genotypes having no or very low heterozygosity; electronic supplementary material, table S1). The number of genotypes maximized available resources in our colony and ensured that the natural distribution of sex-change propensities was represented. We calculated PFSC in each of the 100 genotypes as the proportion of all individuals that transitioned from hermaphrodite to male under common garden conditions in the laboratory colony over a 6.5-year period prior to this study [47]. The current study used animals that were the same number of generations removed from the wild as those for which genotypic variation in PFSC was documented [47]. Across the genotypes used in this study, PFSC ranged from 0 to 68.6%. For genotypes with PFSC < 50%, we used two individuals to ensure against unexpected mortality or sex change in that genotype. For genotypes with PFSC > 50%, we used up to six individuals, assuming that some would change sex but enabling data collection from representatives of those genotypes that did not change sex. All experimental fish were raised from hatchlings in individual plastic containers (Rubbermaid^®^ Take-a-long Deep Squares) filled with approximately 200 ml of 25 parts per thousand (‰) synthetic saltwater (prepared with Instant Ocean^®^ sea salt and aged tap water) until 30 days post hatching (dph), at which time water volume was increased to 600 ml (25‰). At 60 dph, we suspended a large marble-sized ball of egg-laying substrate (Poly-fil^®^) from one top corner of the container. Beginning at 67 dph, each container was checked weekly for eggs in the substrate, around the top edge of the container and on the bottom of the container. Fish were checked every 7 days for eggs until they changed sex to male or were 365−371 dph, ensuring that each remaining hermaphrodite was at least 365 dph and had been checked weekly 43 times.

**Figure 2.**
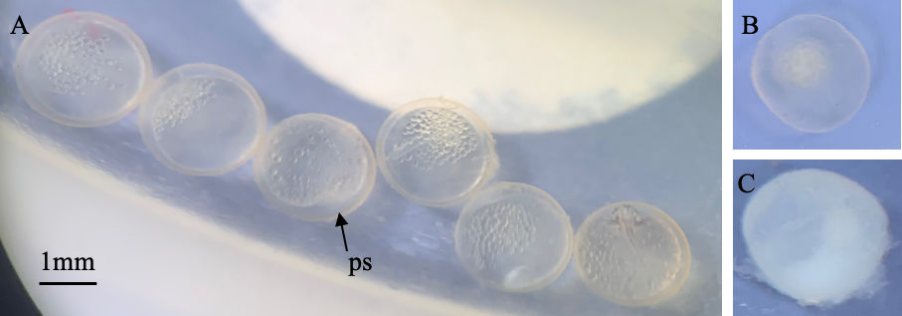
Scoring of eggs. Eggs were scored as (A) fertilized, (B) unfertilized or (C) inviable. Fertilized eggs were identified by the presence of the perivitelline space (ps). Unfertilized eggs lack the perivitelline space. Inviable eggs were identified by their opaque or discoloured appearance.

Eggs were placed into 59 ml plastic cups (Fabri-Kal^®^ Greenware GXL250PC) filled with approximately 6 ml of 25‰ synthetic saltwater. Eggs were immediately counted and viewed under a stereomicroscope. Eggs were photographed and scored as either fertilized, unfertilized or inviable. Fertilized and unfertilized eggs were identified by the presence or absence of a perivitelline space, respectively. Inviable eggs were identified by their marked discoloration and opaque appearance (figure 2). Unfertilized and inviable eggs were removed from the cup and discarded. Fertilized eggs were left in the cups and 6 ml 25‰ water were replaced every 7 days. Cups were checked every day for hatchlings. Hatchlings from the experimental animals were scored as alive or dead, with live hatchlings being euthanized in 4°C salt water. Experimental fish also were observed for signs of sex change each week when the tubs were checked for eggs, as described in Scarsella *et al.* [39]; orange freckles or orange skin is the most reliable external character indicating sex change. Individuals that changed sex (35 of the 213, representing 28 of the 100 genotypes) were separated from the others, and the date and age were recorded. All experimental animals were isolated from the general colony for the duration of the experiment, kept on a 12 h light : 12 h dark photoperiod and fed a 4 ml suspension of brine shrimp (*Artemia*) nauplii (approx. 2000 shrimp) 6 days per week. Room temperature was maintained at 26.45 ± 1.6°C (mean ± s.e.m.). The University of Alabama Institutional Animal Care and Use Committee approved all procedures (IACUC Protocol #18-10-1644).

### (b) Statistical analyses

To determine whether the likelihood of laying eggs within the first 365−371 dph differed among genotypes that varied in their PFSC, we ran a nominal logistic model with ‘laid eggs?’ as a categorical dependent variable (yes = 1, no = 0) and PFSC as the independent variable. We also used a general linear regression model to examine the relationship between fecundity (total number of eggs laid; continuous response) and PFSC (predictor). To determine whether the number of eggs laid per week varied with age, we used a generalized additive mixed model (GAMM) using the R package mgcv [54]. GAMMs use smooth curves to estimate nonlinear effects of factors and are ideal for time series events that exhibit nonlinear fluctuations. In this model, parent ID was a random effect to account for the fact that each parent was checked for eggs many times.

We modelled self-compatibility in two ways: (i) proportion of eggs fertilized and (ii) hatching success of fertilized eggs. To determine whether the proportion of fertilized eggs (response) varied as a function of PFSC (predictor), we ran a general linear regression model. To examine whether the proportion of fertilized eggs varied as a function of the parents’ age when eggs were collected, we again used GAMM with parent ID as a random effect. To ascertain whether the proportion of fertilized eggs (response) varied as a function of fecundity (total number of eggs laid by each parent; continuous predictor), we ran a general linear regression model. We calculated hatch success as the proportion of fertilized eggs that resulted in live hatchlings. To determine whether hatch success (proportion of total eggs that hatched; response) varied as a function of PFSC (predictor), we ran a general linear regression model. To examine whether hatch success varied with parents’ age at egg collection, we used GAMM with parent ID as a random effect (table 1). All analyses and graphing were conducted using R (v. 4.3.2, 2023-10-31 [55]) in RStudio (v. 2024.04.2+764 [56]) using the packages dplyr [57], lubridate [58], tidyr [59], broom [60], car [61], lme4 [62], MuMIn [63], bbmle [64], insight [65], mgcv [54], gam.hp [66], gratia [67] and figures made with ggplot2 [68] and ggthemes [69]. The datasets generated and analysed, as well as the required libraries and notes about model parameters are available in the Figshare repository at: https://figshare.com/projects/Self-Compatibility_Data_Sets_and_Analyses_for_Proceedings_Manuscript/243464.

**Table 1.** Summary of fecundity and self-compatibility models with age of parent fish as the dependent variable. χ^2^ = chi-square value; *p* = *p*‐value; edf = estimated degrees of freedom; de = deviance explained.

fecundity					
model		*χ* ^2^	edf	*p*	de
# eggs ~ age + ID	Age	1147	17	<0.0001	34.7%
	Fish ID	2104	191	<0.0001	65.3%

proportion of eggs that were fertilized					
model		*χ* ^2^	edf	*p*	de
% fertilized ~ age + ID	Age	378.3	6	<0.0001	25.7%
	Fish ID	945.6	138	<0.0001	74.3%

hatch success					
model		*χ* ^2^	edf	*p*	de
hatch success ~ age + ID	Age	82	7	<0.0001	17.6%
	Fish ID	304.9	111	<0.0001	82.4%

## 3. Results

The experiment included 213 individual fish from 100 different genotypes. Thirty-five of those fish changed sex to male (16.4%, age of sex change range: 74−332 dph) before the experiment ended at 365−371 dph. For genotypes in which sex change occurred, the number of individuals that transitioned varied from only one individual per lineage to all individuals. Three of those 35 males (9%) laid eggs before changing sex. We collected 14 310 eggs, had 10 267 hatchlings (71.7% of eggs hatched) and had 10 059 live hatchlings (98% of hatchlings were alive). The three individuals that both laid eggs as hermaphrodites and changed sex to male laid a total of 69 eggs by 197 dph (the oldest age at which these fish were identified as male). This equated to, on average (± standard error), 23 ± 13 eggs per individual among the three, but only 1.97 ± 1.4 eggs *per capita* for the 35 individuals that changed sex to male. The remaining 178 hermaphrodites laid a total of 7341 eggs before they collectively aged to 197 dph (41.2 ± 2.2 eggs per individual). We started checking for eggs from each fish at 67 dph and collected a single egg from one individual on the first check; the egg was fertilized. Overall, 93.4% of the eggs were fertilized, 2.5% were unfertilized and 4.1% were inviable.

**Figure 3.**
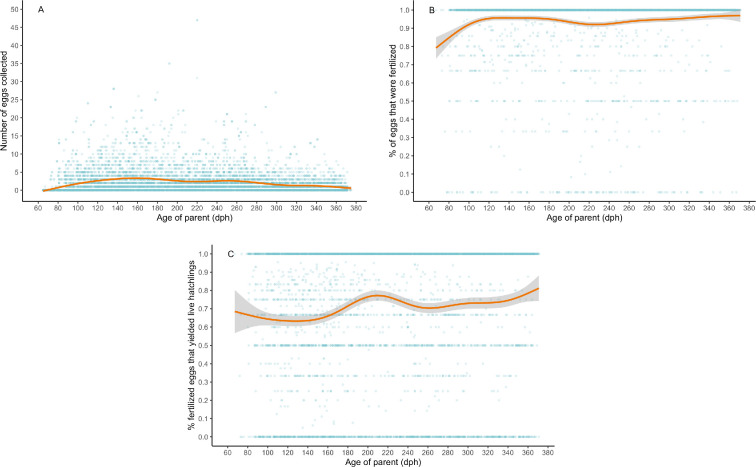
The (A) number of eggs collected across ontogeny (once every 7 days), (B) proportion of those eggs that were fertilized and (C) hatch success of those fertilized eggs. Each point represents the clutch of eggs collected from an individual fish at that age. ‘Deviance explained’ is the proportion of deviance in the response explained by age. Confidence interval bands represent the 95% confidence intervals.

Fecundity (total number of eggs laid), proportion of fertilized eggs and hatching success all varied significantly and nonlinearly with age. Fecundity showed two age-related peaks, one at 160 dph and another at 260 dph, before plateauing around 300 dph and decreasing again at around 365 dph (table 1, figure 3A). The proportion of fertilized eggs increased markedly from 60 to 120 dph, remained constant between 120 and 180 dph, gradually decreased to a nadir at 230 dph and increased again to nearly 100% at 365 dph (table 1, figure 3B). The proportion of fertilized eggs was not associated with clutch size (number of eggs laid in a 7 day period, *F*_1,176_ = 0.468, *p* = 0.495). Hatch success was low for eggs laid by parents aged 60−140 dph, showed a peak for parents aged 210 dph before a dip at 260 dph and a shallow rise until the end of the study period (table 1, figure 3C). In each model, fish ID also was highly significant, indicating consistent differences among individual fish in fecundity and both measures of self-compatibility (table 1).

**Figure 4.**
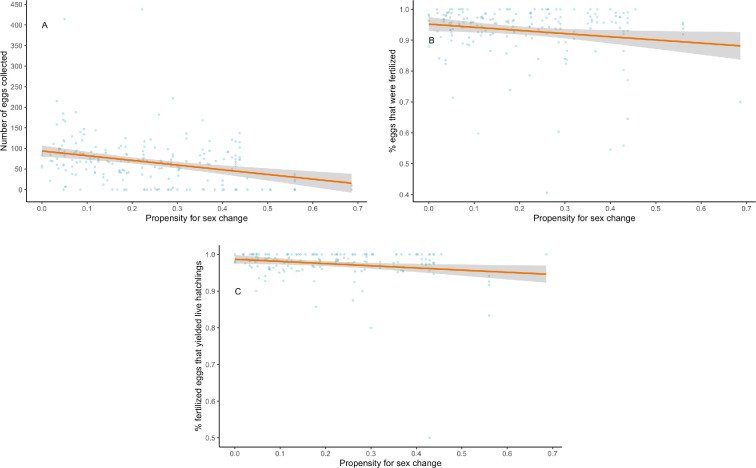
Significant negative relationships between PFSC and (A) fecundity, B) proportion of eggs fertilized and (C) hatch success of fertilized eggs. Each point represents the total or proportion per parent fish. PFSC was measured as the proportion of individuals in each genotype that change sex in isolation under common garden conditions in our colony (lineage % male [47]). Confidence interval bands represent the 95% confidence intervals.

PFSC was significantly associated with measures of both fecundity and self-compatibility. We ran models with and without individuals that changed sex to understand whether and how including hermaphrodites that ultimately opted to forego egg-laying and become male might affect these associations. When sex changers were included, the odds of laying any eggs decreased significantly as PFSC increased (table 2). However, this relationship became non-significant when individuals that changed sex to male were excluded (table 2). The number of eggs laid decreased significantly as PFSC increased, whether or not males were included (table 2, figure 4A). Both the proportion of fertilized eggs and hatch success of those eggs also decreased significantly with increasing PFSC (table 2, figure 4B,C).

**Table 2.** Summary of fecundity, self-compatibility and hatch success models with propensity for sex change (PFSC) as the dependent variable. The models for the proportion of fertilized eggs and hatch success already excluded males that did not lay eggs; thus, separate models were not needed. Significant effects are shown in bold. *F* = *F*-ratio; *z* = *z*-value; *p* = *p*‐value; d.f. = degrees of freedom.

**probability of laying eggs**	**odds ratio**	*z*	d.f.	*p*-value
**PFSC,** ***including*** **males**	**0.003**	**−4.56**	**211**	**<0.0001**
PFSC, *excluding* males	0.006	−1.63	176	0.10

**fecundity (# of eggs laid**)	*estimate*	*F*	d.f.	*p*-value
**PFSC,** ***including*** **males**	**−114.100**	**22.26**	**1, 211**	**<0.0001**
**PFSC,** ***excluding*** **males**	**−68.700**	**6.25**	**1, 176**	**0.0100**

proportion of fertilized eggs	*estimate*	*F*	d.f.	*p*-value
**PFSC,** ***including*** **males**	**−0.103**	**5.13**	**1, 176**	**0.0250**

Hatch success	*estimate*	*F*	d.f.	*p*-value
**PFSC,** ***including*** **males**	**−0.350**	**33.36**	**1, 173**	**<0.0001**

**Figure 5.**
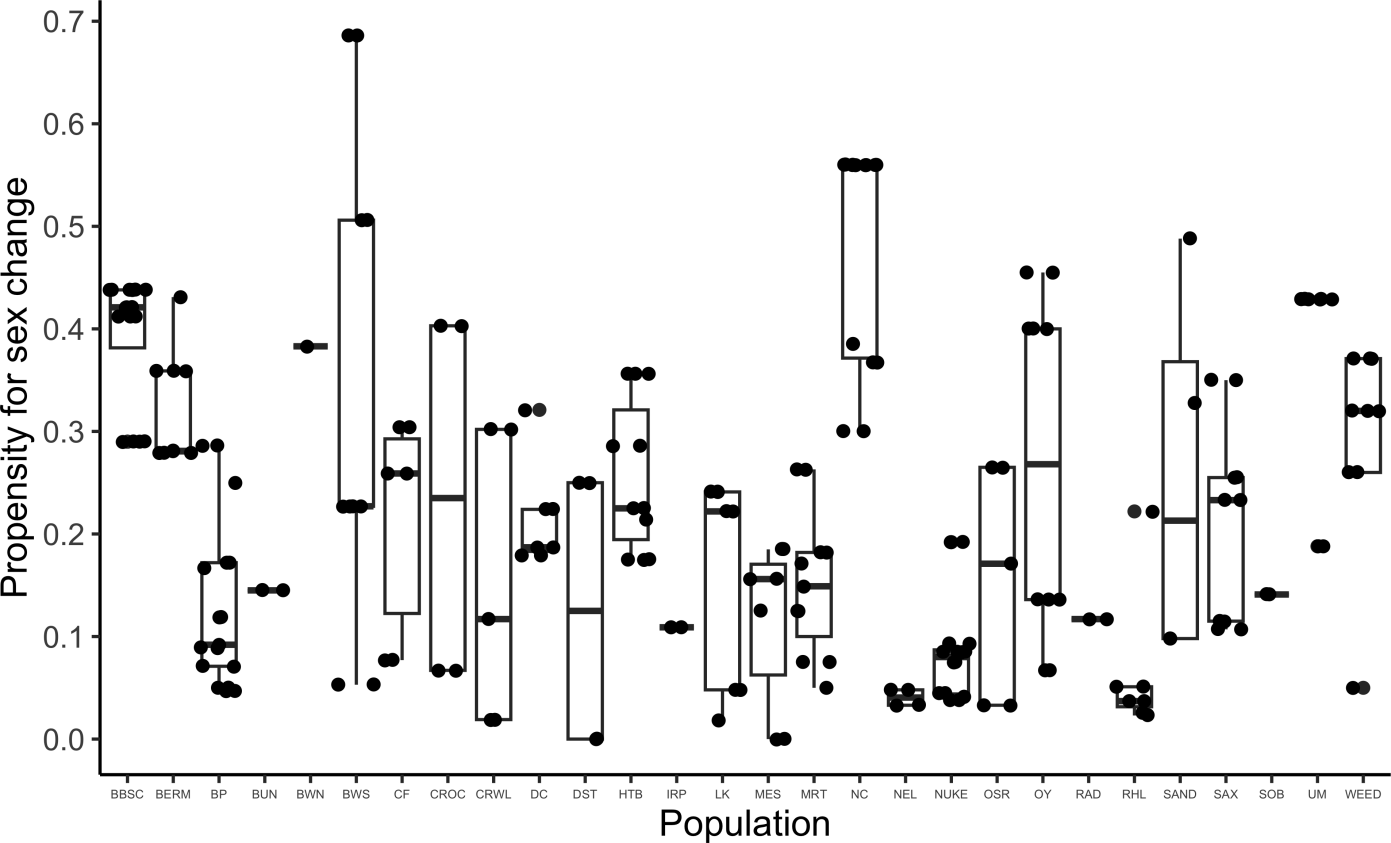
Variation in the propensity to change sex among genotypes within a population and among populations used in this experiment. Each point represents a single genotype in the population. PFSC was measured as the proportion of individuals in each genotype that change sex in isolation under common garden conditions in our colony (lineage % male; [47]).

While most populations maintain considerable genotypic diversity in PFSC (figure 5), eight populations showed very little variation in PFSC (BUN, BWN, IRP, NEL, NUKE, RAD, RHL, SOB). Importantly, we only had one individual from the BWN population and two individuals from the BUN, IRP, RAD and SOB populations, owing to practical constraints of fish availability (e.g. number captured in the field) and genotypic variation within populations.

## 4. Discussion

Mixed-mating strategies offer the fitness rewards of *both* outcrossing and fertilizing one’s own eggs. The cost-to-benefit ratio of adopting strategies along the continuum from completely self-incompatible to completely self-compatible is expected to vary among genotypes, across space and time as the external and internal environments also change, and likely with the interaction between the two (genotype × environment) [1,25–27,70–73]. Genetic variation in self-compatibility, combined with external factors such as pollinator and/or mate availability or parasite pressure, has the potential to either drive or constrain evolutionary changes in mating strategy. For instance, if self-compatible individuals have greater reproductive fitness (i.e. produce more offspring and/or offspring have greater survival rates) and survive just as well as self-incompatible individuals, then the population may evolve to complete or near-complete selfing. Comparatively, if there are plenty of mates (or pollinators) and/or inbreeding depression that increases the benefits of outcrossing, populations will evolve to complete or near-complete outcrossing. Despite these potential fitness differences, whether self-compatibility can evolve and the rate at which it does so ultimately rely on how much variation in self-compatibility exists within a population. Variation in self-compatibility and fecundity in selfing hermaphrodites has been documented only in plants and invertebrates. We tested the hypothesis that self-compatibility and fecundity would vary with age and as a function of genotypic variation in the PFSC in one species from the only self-fertilizing hermaphroditic vertebrate clade (*K. marmoratus* (this study), *K. hermaphroditus*, ‘central clade’ [34]).

Both fecundity and self-compatibility decreased with increasing PFSC, which was the exact opposite of our expectation. We predicted that fecundity and/or self-compatibility would increase with PFSC for multiple non-mutually exclusive reasons: (i) individuals that eventually change sex would be more self-compatible as hermaphrodites and produce more eggs with higher fertilization rates and/or hatch success, and/or (ii) remaining hermaphrodites from high PFSC genotypes would produce at least as many eggs with equal self-compatibility as those produced from low PFSC genotypes. Given that neither of these predictions were supported, it remains unclear how sex change from a selfing hermaphrodite to an obligate outcrossing male is maintained in rivulus populations. While our models showed significant correlations, the *R*^2^ values were quite low and identifying factors responsible for that unexplained variance might provide additional clues. Theory suggests that, compared with self-fertilizing hermaphrodites, males should incur significant costs because they cannot produce offspring on their own and because their genetic contribution to those offspring is halved [1,2]. However, in populations that contain multiple high PFSC genotypes (e.g. Twin Cayes, Belize [50]), individuals that change sex to male coexist with hermaphrodites that are less self-compatible and more likely to lay an unfertilized egg. Such a demographic might increase the reproductive fitness of both males and hermaphrodites.

Males could also overcome the twofold cost by producing many extremely fit offspring. Prior experiments in rivulus have provided evidence for both costs and benefits associated with outcrossing and males. Gresham *et al.* [74] demonstrated outbreeding depression on fecundity traits in several ecologically relevant conditions, *increasing* the cost of both males and outcrossing. However, Ellison *et al.* [75] provided evidence of inbreeding depression through increased parasite stress, therefore *decreasing* the cost of males and outcrossing. Male size also increases with heterozygosity, possibly enabling heterozygous males to secure more resources and greater access to unfertilized eggs, a benefit of outcrossing that is realized when individuals change sex [76]. Clearly, the explanation for the persistence of both outcrossing and of males in rivulus populations remains elusive. Results from the current experiment suggest that the cost of being male is highly variable among genotypes and populations. Over the 43 week period, only 3 of 35 individuals that changed sex laid eggs—sex changers were not as fecund as self-fertilizing hermaphrodites that never switched. Sex changers that laid eggs showed a 44% reduction in egg production relative to those that did not change sex. Given their low fecundity, the reproductive cost of changing sex to male is likely low for those individuals, but those males must find unfertilized eggs and produce offspring whose fitness is equal to (or higher than) the fitness of selfed offspring to prevent sex-changing alleles from going extinct. Currently, we have no conclusive evidence that either of these conditions is true.

When all individuals were analysed, the odds of laying *any* eggs decreased with increasing PFSC. However, when we excluded individuals that changed sex, there was no relationship between PFSC and egg-laying odds, so hermaphrodites from high PFSC genotypes are just as likely to lay *an* egg as those from low PFSC genotypes. In contrast, hermaphrodites from high PFSC genotypes laid significantly *fewer* eggs, even when sex changers were excluded. Self-compatibility and hatch success also decreased as PFSC increased. These results show that individuals from high PFSC genotypes that remain hermaphrodites are not very fecund or self-compatible, supporting the idea that the costs of sex change vary among genotypes. Individuals from low PFSC, highly fecund and self-compatible genotypes are likely to experience the highest cost of sex change, as they would trade high reproductive success as a hermaphrodite for very low reproductive success as a male. This cost would increase in populations containing mostly low PFSC genotypes (e.g. NEL, NUKE, RHL; figure 5) as very few unfertilized eggs would be available for outcrossing. Conversely, hermaphrodites from high PFSC genotypes are less fecund and self-compatible anyway, thus reducing the cost of sex change. Any individual that changes sex to male in a population of high PFSC genotypes (significantly lower self-compatibility) may benefit from a greater number of unfertilized eggs (e.g. NC; figure 5).

Regardless of the cost of changing sex to male, there is still the issue of passing sex-changing alleles on to the next generation. How do high PFSC genotypes that are less fecund and less self-compatible not disappear from populations? It is possible that many of these genotypes do go extinct, but some Belizean populations maintain relatively high male proportions (>20%) and non-trivial rates of outcrossing [38,50,77,78], so males and outcrossing appear to be under positive selection in some places. Males must be able to successfully find unfertilized eggs, but we are not sure how that happens. Mackiewicz *et al.* [52] paired multiple senescent hermaphrodites with males in individual tubs and confirmed that 2 out of 32 offspring were outcrossed progeny. Nakamura *et al.* [79] surgically removed eggs from two hermaphrodites for *in vitro* outcrossing, but 12 of the 13 hatchlings were self-fertilized. We have also tried pairing hermaphrodites and males in different ratios with no outcrossing success (RE, 2013 - 2014). Reported interactions between pairs of hermaphrodites and males have ranged from no courtship behaviours [74,80] to significantly more courtship behaviours than same-sex pairs [81] or courtship behaviours by males and reciprocal attacks by hermaphrodites [50]. Thus, we have yet to reproducibly identify conditions that favour outcrossing.

There often are opposing selection pressures for and against biparental sex (outcrossing). In mixed-mating systems, individuals can benefit from outcrossing when the costs of biparental sex are low, but self when the costs of biparental sex are high or when mates and/or pollinators are sparse. The relative benefit of selfing or outcrossing also depends on individual self-compatibility, which can have a genetic basis and/or respond to the external environment in a plastic or flexible manner [25–27,29]. Less self-compatible individuals and genotypes cannot exploit the benefits of selfing, even when the costs of outcrossing are high. In populations with few or no genotypes that frequently change sex (e.g. those recently founded by highly self-compatible individuals), genotypes with higher PFSC would likely struggle to establish, even if offspring derived from selfing and outcrossing are equally fit. This is because individuals from high PFSC genotypes that transition to male will find very few opportunities (unfertilized eggs) to pass their sex-changing alleles on to the next generation. However, if high PFSC genotypes become established, possibly owing to additional migration or outcrossed progeny having a fitness advantage over selfed progeny, sex change (and by definition, males) would be maintained in the population. In this study (figure 5) and in Gresham *et al.* [47], we found genotypic variation in PFSC within populations, but some populations appear to maintain a much greater range of PFSC. Indeed, across rivulus’ geographic range, we find significantly higher outcrossing rates in populations with more high PFSC genotypes [37,78].

Males, and genotypes that frequently produce males, may also have better success in populations with many young hermaphrodites. While we did not find evidence that younger hermaphrodites reproduce as ‘pure females’ and only lay unfertilized eggs [49], younger hermaphrodites laid *more* unfertilized eggs. We discuss the age-dependent variation of fecundity and self-compatibility in greater detail in the electronic supplementary material. Some of the variation in egg-laying, especially the decline from 300 to 371 dph, could be owing to reproductive seasonality retained under laboratory conditions [43]. If the fitness of progeny derived from selfing and outcrossing is relatively equal, population age structure can select for or against high PFSC genotypes. Populations in which juvenile and young adult mortality are high will have proportionally fewer unfertilized eggs available for males than populations in which younger individuals thrive, selecting against high PFSC genotypes. These results open fascinating opportunities for future studies on how fitness varies with genotypic variation in size, fecundity and PFSC across different environments, and for field studies that explore putative selection pressures (e.g. parasites, predation, competition) that might drive sex ratio variation across populations and that track demographic and population genetic characteristics such as selfing and outcrossing rates, proportion of males, population size and density.

## Data Availability

The datasets generated and analysed, as well as the required libraries, and notes about model parameters are available in the Figshare repository [82]. Supplementary material is available online [83].
